# A meta-analysis of the efficacy of physical exercise interventions on activities of daily living in patients with Alzheimer's disease

**DOI:** 10.3389/fpubh.2024.1485807

**Published:** 2024-11-27

**Authors:** Yang Xiao, Yu Fan, Zhengteng Feng

**Affiliations:** ^1^College of Physical Education and Sports, Beijing Normal University, Beijing, China; ^2^Department of Physical Education, Nanjing University of Science and Technology, Nanjing, China; ^3^China Athletics College, Beijing Sport University, Beijing, China

**Keywords:** Alzheimer's disease, physical exercise, activities of daily living, intervention effect, meta-analysis

## Abstract

**Objective:**

This study aimed to systematically review published randomized controlled trials on the effects of physical exercise on activities of daily living (ADL) in Alzheimer's patients through meta-analysis, thereby synthesizing existing evidence to provide scientific intervention recommendations for exercise prescriptions in Alzheimer's patients.

**Methods:**

Based on strict literature inclusion and exclusion criteria, a systematic search was conducted in databases including PubMed and Web of Science from their inception to July 1, 2024. The Cochrane risk assessment tool was used to evaluate the design of randomized controlled trials. Studies reporting on physical exercise interventions for ADL in Alzheimer's patients were systematically identified. Subgroup analyses and meta-regression were performed to explore sources of heterogeneity.

**Results:**

Nineteen articles, for analysis, providing 27 randomized controlled trials (RCTs). A random-effects model was used to calculate the effect size and 95% confidence interval for each independent study, and meta-analysis was performed using Stata 16.0 and RevMan 5.4 software. The results showed that physical exercise might significantly improve ADL in Alzheimer's patients (SMD = 0.33, 95% CI: 0.12–0.54, *I*^2^ = 81.7%). Sensitivity analysis confirmed the robustness of the results (*p* > 0.05). Egger's test did not reveal significant publication bias (*p* = 0.145). Samples were divided into different subgroups based on intervention content, duration, frequency, and session length. Subgroup analysis based on intervention characteristics showed that resistance training or aerobic exercise (SMD = 0.83, 95% CI: 0.60–1.05), long-term interventions (>6 months, SMD = 0.31, 95% CI: 0.13–0.49), medium-frequency interventions (4-5 times per week, SMD=0.39, 95% CI: 0.23-0.55), and short-duration training ( ≤ 30 min, SMD = 0.96, 95% CI: 0.71–1.21) might be most effective in enhancing ADL in Alzheimer's patients. These improvements were not only statistically significant but also had substantial impact in clinical practice.

**Conclusion:**

Resistance training or aerobic exercise lasting more than 6 months, 4–5 times per week, and lasting no more than 30 min per session may be most effective in improving ADLs in patients with Alzheimer's disease.

## 1 Introduction

Alzheimer's disease (AD) is a progressive, irreversible neurodegenerative disorder that severely impacts patients' quality of life. As China's population continues to age, the number of individuals with cognitive impairment is steadily increasing. Currently, dementia patients account for over 5% of China's population, representing ~25% of the global dementia population (1). AD is the most common cause of cognitive impairment, accounting for about 60% of all dementia cases (2). The pathogenesis of AD remains unclear, but it may be associated with cholinergic dysfunction, genetic variations, tau protein abnormalities, and abnormal aggregation of amyloid β (Aβ). At present, there is no effective treatment targeting the underlying causes of AD. In 2011, the new National Institute on Aging-Alzheimer's Association (NIA-AA) criteria (3) expanded the concept of AD to include three stages: preclinical, mild cognitive impairment (MCI), and dementia, aiming to develop systematic treatment approaches for AD.

The current standard treatment primarily involves pharmacological interventions. However, drug therapies for AD have limited efficacy and significant side effects. No curative medication exists for AD, and clinical management focuses on symptomatic treatment. Cognitive-enhancing drugs and antipsychotics have limited effectiveness for AD patients, who often experience treatment inefficacy, adverse effects, and tolerance issues (4–6). Physical exercise, defined as interventions based on kinesiology, biomechanics, and neurodevelopmental principles, aims to improve physical, mental, and cognitive functional impairments through the application of force and counterforce. As a non-pharmacological treatment, physical exercise is widely applied to various diseases. Existing research indicates that non-pharmacological interventions can effectively prevent and delay cognitive decline (7). Numerous clinical trials on physical exercise for AD treatment have yielded positive results. However, a detailed assessment of the potential benefits of physical exercise for AD patients to validate its recommendation or clinical application is lacking. Although some studies have suggested that physical exercise may be associated with improved activities of daily living (ADL) in AD patients, there is a lack of systematic reviews synthesizing evidence in this area. The most recent systematic review on this topic dates back to 2023, and a substantial body of literature has been published since then. In light of this, our study collects randomized controlled trials (RCTs) of physical exercise interventions in AD patients for systematic evaluation and proposes exercise prescriptions. We aim to provide evidence-based guidance for physical exercise interventions in AD patients and offer references for the prevention and clinical treatment of AD in middle-aged and older adult populations.

## 2 Research methods

### 2.1 Literature search

This study employed a computerized search of databases including PubMed, Web of Science, ScienceDirect, EMbase, and Cochrane Library. The search period covered from the inception of each database to July 2024, adhering to the Preferred Reporting Items for Systematic Reviews and Meta-Analyses (PRISMA) guidelines for literature retrieval. The search strategy combined subject headings and free-text terms, underwent repeated preliminary testing, and was supplemented by manual searching to systematically include randomized controlled trials.

The search was guided by the PICO model to ensure a structured approach. The Population (P) included older adult patients with Alzheimer's disease. The Intervention (I) was any form of physical exercise, and the Comparison (C) was against control groups receiving no exercise or alternative interventions. The Outcome (O) was the improvement in Activities of Daily Living (ADL), instrumental activities of daily living (IADLs), and other scales that measure functional abilities. This approach allowed us to ensure that the studies selected for inclusion were directly relevant to the research question and aligned with the PICO framework.

The search focused on several key concepts: randomized controlled trials to ensure the inclusion of high-quality experimental studies, physical exercise interventions, Alzheimer's disease and its various terminologies, including dementia and related neurodegenerative conditions, as well as functional assessments related to Activities of Daily Living (ADL), instrumental activities of daily living (IADLs), and other scales that measure the ability to perform daily tasks. This structured search approach was developed following PRISMA guidelines, ensuring the broadest possible inclusion of studies evaluating the effects of physical exercise interventions on ADL in patients with Alzheimer's disease.

### 2.2 Literature inclusion and exclusion criteria

#### 2.2.1 Literature inclusion criteria

**Figure 1:**
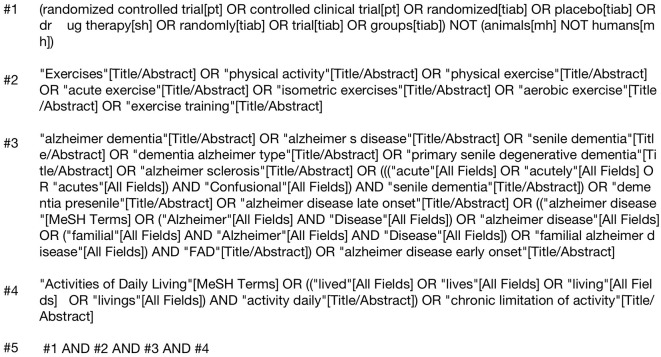
PubMed search strategy.

This study included randomized controlled trials (RCTs) focusing on Alzheimer's disease patients (Figure 1). The inclusion criteria were as follows: (1) Study type: Randomized controlled trials (RCTs). (2) Study subjects: Older adult dementia patients aged 60 years and above, diagnosed according to the Chinese Classification and Diagnostic Criteria of Mental Disorders (CCMD-3) or the Diagnostic and Statistical Manual of Mental Disorders, Fourth Edition (DSM-IV). Patients must have undergone head CT or MRI within 1 year prior to enrollment, with results consistent with an AD diagnosis. (3) Comparability: The intervention and control groups should be comparable in terms of general demographic data, disease condition, and course of illness. There should be no statistically significant differences in the observed indicators between the two groups prior to intervention. (4) Outcome measures: Results should include scores from the Activities of Daily Living (ADL) scale, Basic Activities of Daily Living (BADL) assessment scale, Alzheimer's Disease Cooperative Study—Activities of Daily Living (ADCS-ADL) scale, or Instrumental Activities of Daily Living (IADL) scale. Mean values and standard deviations for pre- and post-intervention periods should be provided.

#### 2.2.2 Exclusion criteria

This study excluded the following literature: (1) Observational studies without a control group. (2) Duplicate publications or data. (3) Incomplete original data or inability to extract necessary data from the literature. (4) Studies that did not provide detailed intervention methods or where interventions were limited to home or hospital settings only. (5) Studies that did not clearly specify the duration of intervention. (6) Studies not focused on Alzheimer's disease patients.

### 2.3 Literature screening and data extraction

**Figure 2:**
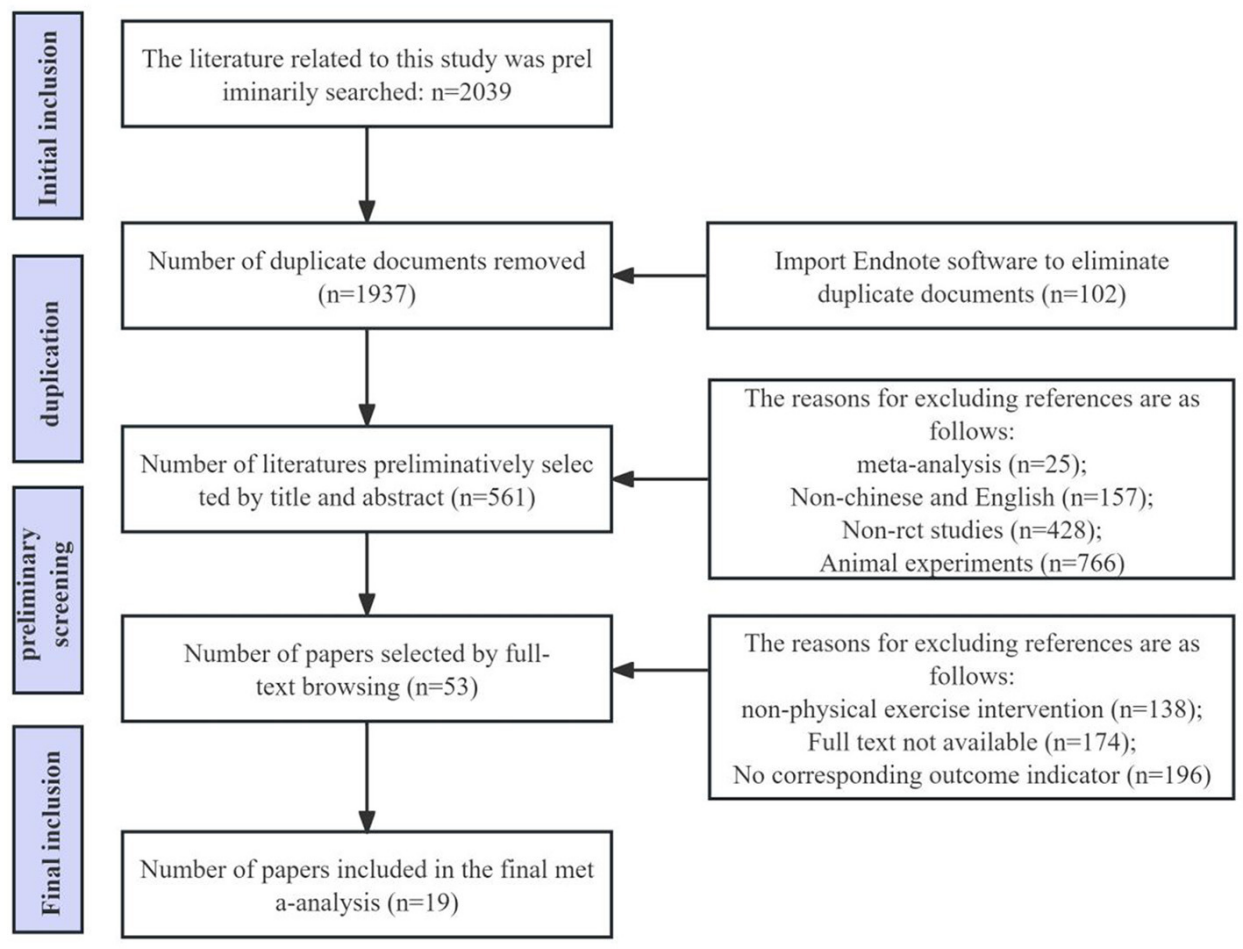
Literature screening flow chart.

This study strictly adhered to the PRISMA statement and Cochrane Handbook for Systematic Reviews of Interventions during the literature screening and data extraction process. Initially, two researchers independently searched literature in databases such as PubMed, Web of Science, and Cochrane Library according to the predetermined search strategy. The search results were imported into EndNote X9 software for management and deduplication. Subsequently, the two researchers screened the literature based on inclusion and exclusion criteria, conducting an initial screening (reading titles and abstracts) and a secondary screening (reading full texts). After completing both screenings, the two researchers cross-checked their results, resolving any disagreements through discussion or arbitration by a third researcher. The specific literature screening process is illustrated in the PRISMA flow diagram (Figure 2).

For the included literature, two researchers independently extracted the following information using a standardized data extraction form: (1) study characteristics (title, first author, publication year, country, study design, follow-up time); (2) subject characteristics (sample size, age, menopausal time, baseline bone density); (3) intervention measures (exercise type, frequency, intensity, duration, resistance level, etc.); (4) control group treatment; (5) outcome indicators (ADL, BADL, ADCS-ADL, IADL).

Data synthesis and meta-analysis were performed using RevMan 5.4 software. The *I*^2^ statistic was used to assess heterogeneity among included studies, with *I*^2^ < 50% indicating low heterogeneity and the use of a fixed-effects model, otherwise a random-effects model was applied. Sources of heterogeneity were explored through subgroup analysis and meta-regression. Sensitivity analysis was used to evaluate the impact of individual studies on the overall effect. Egger's test and funnel plots were used to assess publication bias. All statistical tests were set at *P* < 0.05 for statistical significance. The entire process of data extraction and analysis strictly followed the predetermined protocol, completed independently by two researchers in a double-blind manner. Literature selection through title and abstract review was cross-checked, with disagreements between the two evaluators resolved through discussion and third-party review, ensuring the accuracy and reliability of the results.

### 2.4 Literature quality assessment

**Figure 3:**
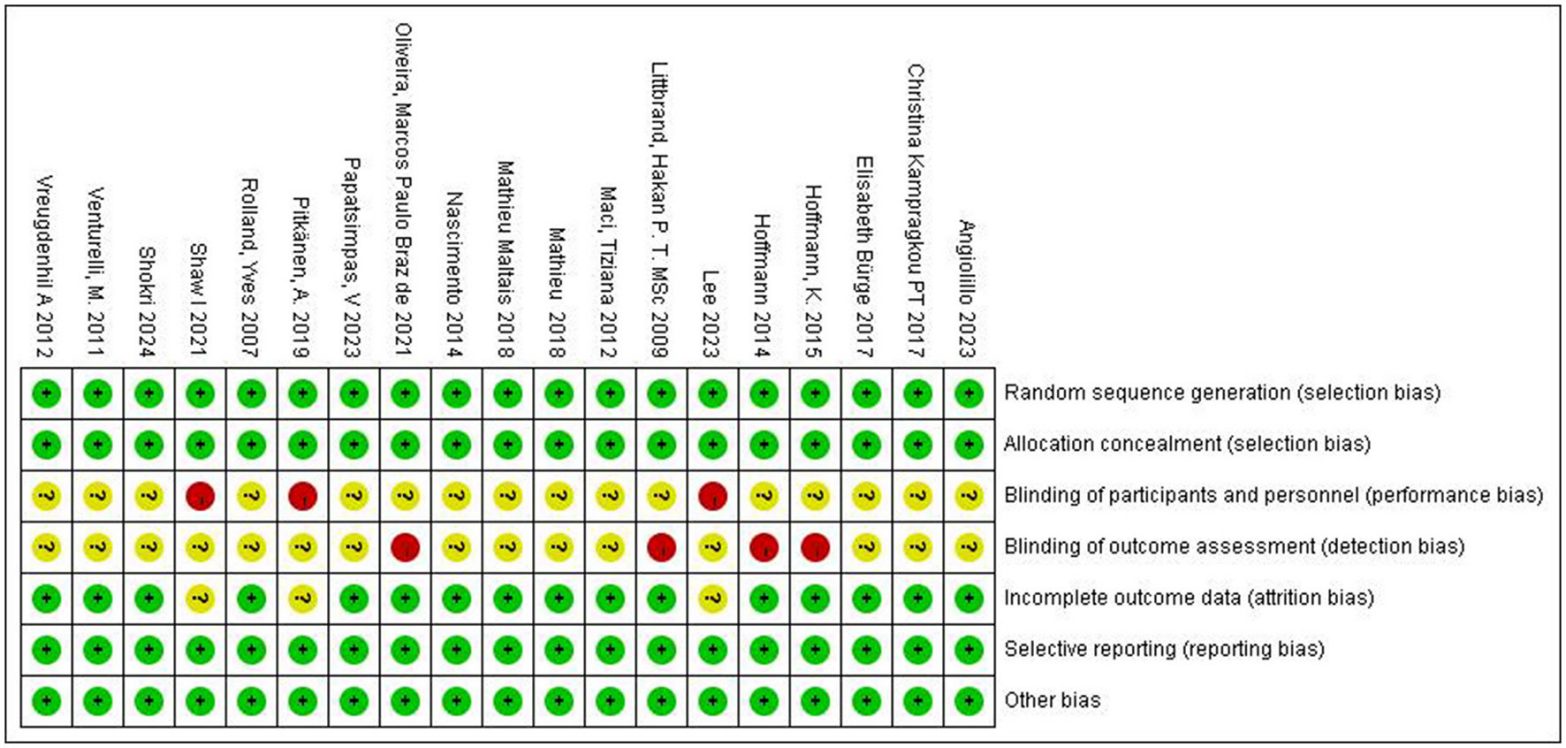
Assessment of the risk of bias for included studies. +, low bias risk; –, high bias risk; ?, unclear bias risk.

To evaluate the methodological quality of included studies and identify potential risks of bias, we used the risk assessment tool from the Cochrane Handbook for Systematic Reviews of Interventions version 6.2. The assessment included: (1) generation of random sequence; (2) blinding of participants, researchers, and outcome assessors; (3) allocation concealment; (4) completeness of outcome data; (5) selective reporting of study results; and (6) other sources of bias. The assessment results for each item were classified into three levels: “low risk of bias,” “high risk of bias,” and “unclear risk of bias” (Figure 3).

Two researchers independently assessed the risk of bias for each study, recording the results using Review Manager 5.4 software. Finally, the two researchers exchanged assessment results, resolving any inconsistencies through discussion or arbitration by a third researcher.

**Figure 4:**
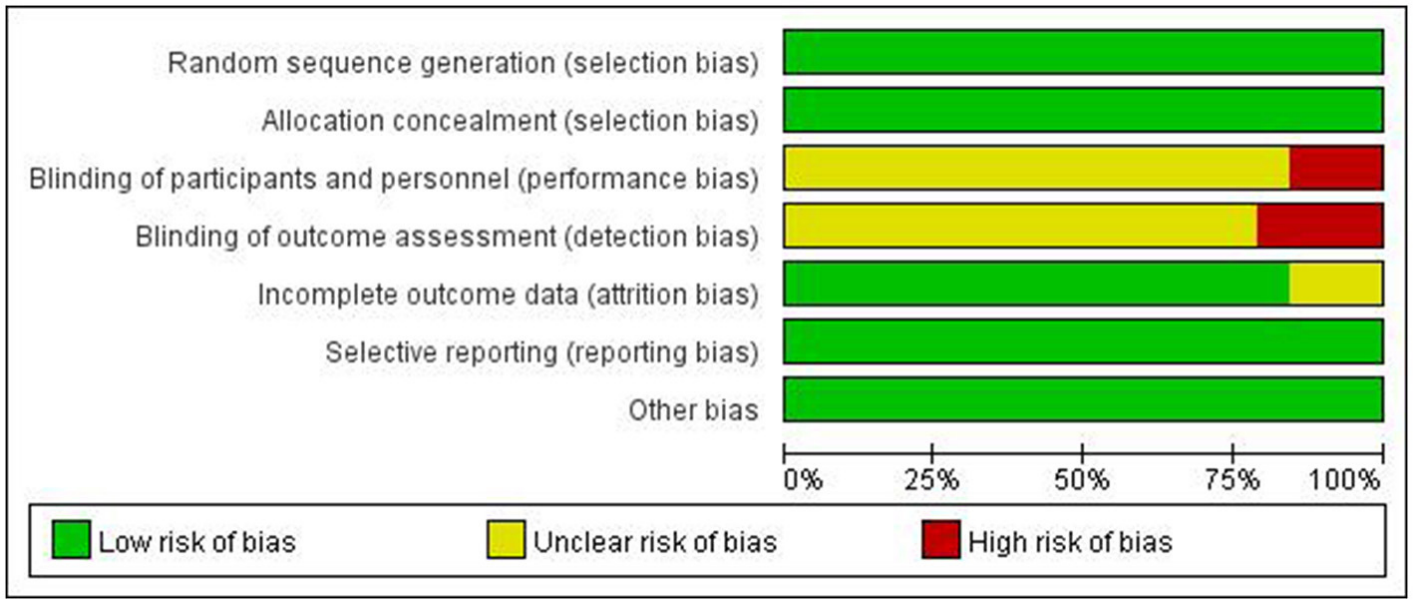
Distribution of studies across the bias ranking for each type of bias.

A risk of bias assessment graph was created after summarizing the results for all included studies (Figure 4). This graph visually displays the assessment results for each study across various sources of bias, using different colors to represent different levels of bias risk: green for low risk, yellow for unclear risk, and red for high risk. For studies with high or unclear risk of bias, we considered these factors when interpreting meta-analysis results and conducted subgroup or sensitivity analyses when necessary.

The literature quality assessment process strictly followed the standard methods in the Cochrane Handbook to ensure the standardization and objectivity of the assessment. The use of high-quality literature provided a reliable evidence base for subsequent meta-analysis.

### 2.5 Basic characteristics of included literature

This study included 19 articles, comprising 27 randomized controlled trials (RCTs), with 870 participants in the experimental group and 830 in the control group. There were no statistically significant differences between the two groups in terms of age, weight, and other aspects (*p* > 0.05), ensuring comparability. Among them, 280 participants were in the multi-component training group, 223 in the aerobic exercise group, 56 in the low-intensity training group, 220 in the strength, walking, and balance training group, and 91 in the resistance training or aerobic exercise group. The mean age of the study subjects was 74.7 years. ADL, BADL, ADCS-ADL, and IADL were used as outcome indicators. Details of exercise intervention cycles, intervention frequency, and single intervention duration are presented in Table 1.

**Table 1:** Features of the literature included in the study.

**References**	**Sample size (T/C)**	**Age**	**Sports intervention characteristics**				**Outcome**	**Follow-up visit**
			**Content**	**Cycle (month)**	**Frequency (times/week)**	**Duration (minutes/ time)**		
Maltais et al. (8)	40/45	≥65	Multi-component training	6	2	60		12
Hoffmann et al. (9)	102/88	≥50	Moderate to high intensity aerobic exercise	4	3	60		–
Maci et al. (10)	7/7	≥60	Balance, gait, eye-hand, coordination, breathing exercises	3	5	60		–
Nascimento et al. (11)	14/16	≥70	Multimodal aerobic	6	3	60		–
Rolland et al. (12)	56/54	≥75	Cardio, strength, flexibility and balance	12	2	60		6
Pitkänen et al. (13)	86/89	≥65	Balance, flexibility, strength training	NA	3	30–60		–
Vreugdenhil et al. (14)	20/20	≥50	Resistance training or cardio	6	3	30–60		–
de Castro Cezar et al. (15)	16/19	≥65	Strength, balance	4	3	60		–
Shaw et al. (16)	14/20	≥70	Balance, resistance, aerobic and flexibility	2	3	45		–
Kampragkou et al. (17)	15/15	≥65	Cardio combined with music therapy and memory training	3	3	45		–
Venturelli et al. (18)	11/10	≥75	Walk	6	4	30		–
Littbrandet al. (19)	43/48	≥65	Functional weight bearing	3	3	45		–
Bürge et al. (20)	78/82	≥70	Strength, walking and balance training	1	5	30		–
Shokri et al. (21)	41/14	50–75	Remote sports training with music	3	3	35–45		–
Hoffmann et al. (9)	107/93	50–0	Moderate to high intensity aerobic exercise	4	3	60		–
Papatsimpas et al. (22)	57/57	≥65	Combine aerobic and resistance exercises	1	5	30–45		–
Lee et al. (23)	140/140	71	Multi-component training	10	1	90		–
Angiolillo et al. (24)	9/1	NA	Strength, walking and balance training	6	2	60		–
Mendes et al. (25)	12/14	≥65	Multi-component training	1	3	60		–

T stands for experimental group; C stands for control group; represents ADCS-ADL; Indicates the IADL. means BADL; denotes ADL; “–” indicates that the value is missing.

### 2.6 Statistical analysis

This study followed the PRISMA 2020 reporting guidelines. Meta-analysis was performed using Stata16.0 software, with standardized mean difference (SMD) as the effect size, accompanied by 95% confidence intervals (95% CI) to describe the effect size of each study for continuous outcome indicators (such as IADL). Before combining meta-analyses, heterogeneity tests were conducted using the Homogeneity test (test level a = 0.1), i.e., χ^2^ test. *p* < a indicated heterogeneity among studies; otherwise, studies were considered homogeneous. The magnitude of heterogeneity was quantitatively analyzed using *I*^2^, with *I*^2^ < 25% considered as low heterogeneity, 25–50% as moderate heterogeneity, and >50% as high heterogeneity. If *I*^2^ < 50% and *p* > 0.05, heterogeneity among studies was considered acceptable, and a fixed-effects model was used for analysis; if *I*^2^ ≥ 50% and *p* ≤ 0.05, heterogeneity among studies was considered significant, and a random-effects model was used for analysis.

## 3 Meta-analysis results

### 3.1 Heterogeneity test

**Figure 5:**
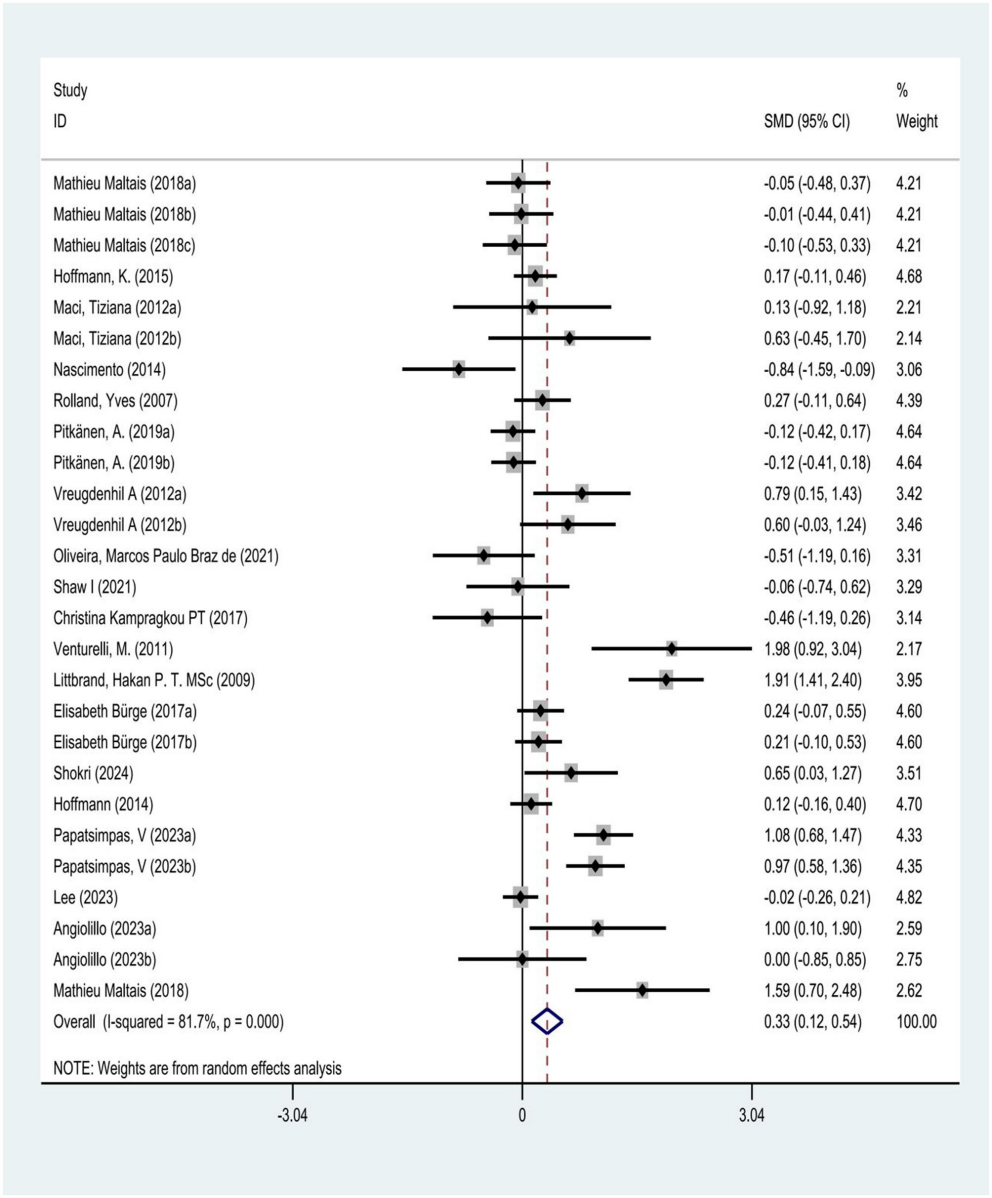
Heterogeneity analysis of physical exercise on daily living ability of AD patients.

This study pre-set the statistical threshold for heterogeneity assessment at *I*^2^ >50% to determine the presence of heterogeneity, using the *I*^2^ statistic to test heterogeneity among studies. The results showed *I*^2^ = 81.7% > 50%, *P* = 0.00 < 0.05, with a total effect size of 0.33, 95% CI (0.12, 0.54), indicating statistically significant heterogeneity among the selected literature. According to the heterogeneity magnitude criteria, an *I*^2^ value between 50 and 75% indicates substantial heterogeneity. The heterogeneity may stem from differences in study subject characteristics, exercise intervention duration, and outcome measurements (Figure 5).

### 3.2 Sensitivity analysis

**Figure 6:**
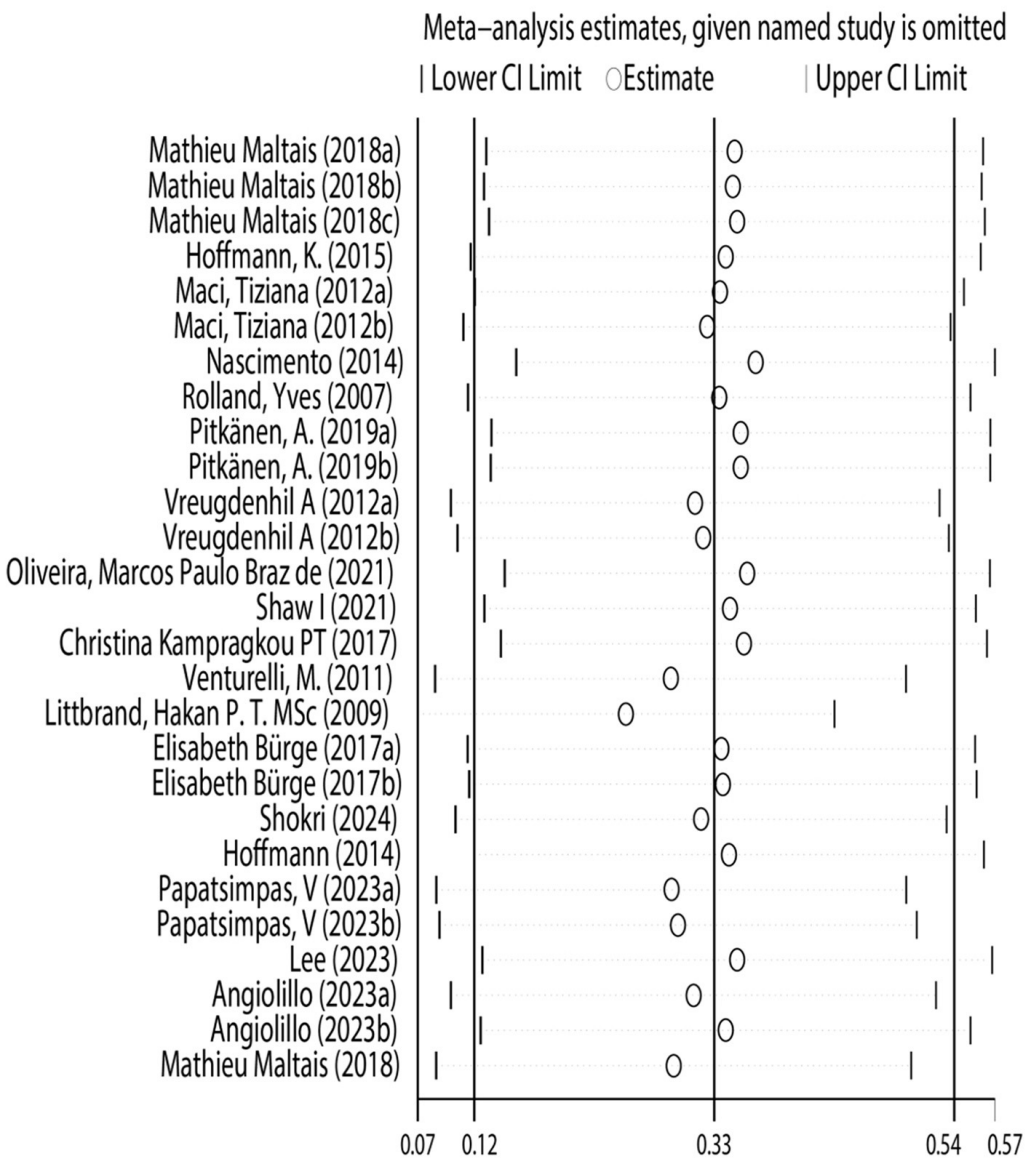
Sensitivity analysis.

This study conducted sensitivity analysis to verify the stability and reliability of the research results. Using the leave-one-out method, each study was excluded in turn, and the pooled effect size was recalculated. The results showed that regardless of which study was excluded, the impact on the results was minimal, with little change in the pooled effect size and confidence interval. This verified the robustness of the study's conclusions, confirming that the conclusion regarding the effectiveness of physical exercise as an intervention measure for improving the risk of daily living ability in Alzheimer's patients is reliable (Figure 6).

### 3.3 Publication bias analysis

**Figure 7:**
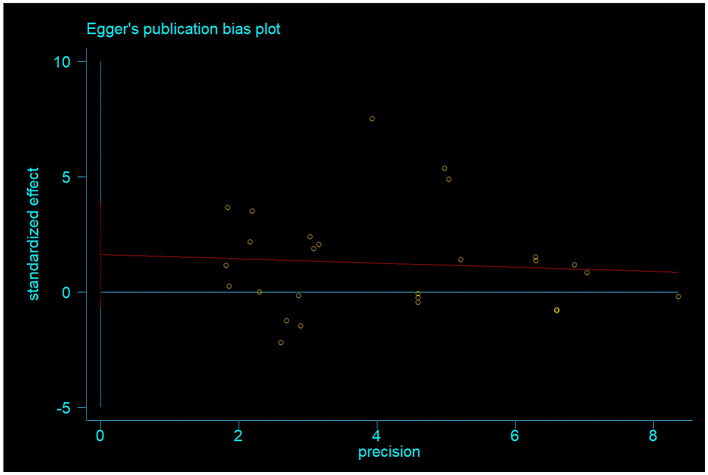
Egger method tests publication bias.

To test for publication bias, Egger's test was used to analyze the included studies. The results showed that the *p*-value of Egger's test was 0.145 > 0.05. Additionally, the funnel plot drawn in this study showed a relatively symmetrical distribution of literature, with basically symmetrical scatter points on both sides. This indicates that there is no publication bias in the meta-analysis results of physical exercise as an intervention measure for improving the risk of daily living ability in Alzheimer's patients (Figure 7).

### 3.4 Meta-regression analysis

To assess the impact of different exercise prescription parameters (intervention content, intervention cycle, intervention frequency, and duration of each intervention) on the effect size, a random-effects model was used for meta-regression analysis. The results showed no statistically significant effects on the daily living ability of Alzheimer's patients for intervention content (regression coefficient = 0.12, 95% CI −0.89 to 1.10, *P* = 0.81), intervention cycle (regression coefficient = 0.23, 95% CI −0.32 to 0.77, *P* = 0.40), intervention frequency (regression coefficient=0.55, 95% CI 0.67–1.04, *P* = 0.03), or duration of each intervention (regression coefficient = 0.91, 95% CI 0.20–1.63, *P* = 0.02) (Table 2). Considering the heterogeneity in the included literature, it was not possible to determine the optimal exercise prescription from the meta-analysis results. To obtain clearer evidence, pre-planned subgroup analyses were conducted to compare the differences in improvement effects on the risk of daily living ability in Alzheimer's patients among different exercise parameter subgroups, providing optimized recommendations for physical exercise for Alzheimer's patients' daily living ability.

**Table 2:** Meta-regression analysis of the intervention effect of physical exercise on the ability of daily living in Alzheimer's patients.

**_ES**	**Coef**.	**Std. err**.	***t***	***P* > *t***	**(95% conf**.	**interval)**
interventioncontent	0.12	0.48	0.24	0.81	−0.89	1.10
interventioncycle	0.23	0.26	0.86	0.40	−0.32	0.77
interventionfrequency	0.55	0.24	2.35	0.03	0.67	1.04
length of each intervention session	0.91	0.35	2.62	0.02	0.20	1.63
outcome	0.12	0.64	0.18	0.86	−1.20	1.44

### 3.5 Subgroup analysis

To further explore the impact of physical exercise on the risk of daily living ability in Alzheimer's patients, pre-planned subgroup analyses were conducted. First, based on the intervention content for Alzheimer's patients' daily living ability, the experimental group was divided into multi-component training; aerobic exercise; low-intensity training; strength, walking, and balance training; and resistance training or aerobic exercise. The results showed that compared with the control group, all five subgroups could significantly improve the risk of daily living ability in Alzheimer's patients, with statistically significant differences. Among them, resistance training or aerobic exercise (SMD = 0.83, 95% CI 0.60–1.05) may have a more positive impact on preventing the risk of daily living ability in Alzheimer's patients. Second, according to the intervention cycle, the sample was divided into short-cycle intervention group (< 6 months), medium-cycle intervention group (6 months), and long-cycle intervention group (>6 months). It was found that the long-cycle group (SMD = 0.31, 95% CI 0.13–0.49) showed better improvement in fracture risk (*p* < 0.05), and the differences between the short-cycle, medium-cycle, and long-cycle groups and the control group were statistically significant, showing significant positive effects (*p* < 0.01). This suggests that long-cycle exercise interventions may be more beneficial in preventing the risk of daily living ability in Alzheimer's patients. Third, based on intervention frequency, the sample was divided into low-frequency intervention group (< 3 times/week), medium-frequency intervention group (4–5 times/week), and high-frequency intervention group (>5 times/week and above). The medium-frequency intervention group (SMD = 0.39, 95% CI 0.23–0.55) may have a more positive impact on the daily living ability of Alzheimer's patients (*p* < 0.05). Fourth, according to the duration of each intervention, the sample was divided into short duration ( ≤ 30 min), medium duration (31–60 min), and long duration (>60 min and above). The results showed that the short duration intervention subgroup (SMD = 0.96, 95% CI 0.71–1.21) was superior to the other two groups. Finally, based on outcome indicators, the sample was divided into ADCS-ADL group, IADL group, BADL group, ADL group, and ADAS-Cog group. The results showed that the IADL group (SMD = 0.36, 95% CI 0.22–0.51) may have the best effect in assessing the risk of daily living ability in Alzheimer's patients, with a significant impact (*p* < 0.01) (Table 3).

**Table 3:** Subgroup analysis of the intervention effect of physical exercise on daily living ability of Alzheimer's patients.

**Group**		**Number**	**SMD**	**95%CI**		***P***	***I*^2^/%**	**The *p*-value of SMD**
Intervention content	Multi-component training	6	−0.01	−0.16	0.13	0.023	61.80%	0.843
	Aerobic exercise	3	0.08	−0.11	0.27	0.044	68.10%	0.422
	Strength, walking and balance training	11	0.30	0.16	0.44	0	85.00%	0.000
	Resistance training or cardio	5	0.83	0.60	1.05	0.061	55.60%	0.000
	Low-intensity training	2	0.18	−0.29	0.65	0.022	80.80%	0.456
Intervention cycle	Short cycle	13	0.27	0.16	0.37	0	77.70%	0.000
	Medium cycle	7	0.04	−0.14	0.22	0	80.70%	0.675
	Long cycle	7	0.31	0.13	0.49	0	88.50%	0.001
Intervention frequency	Low frequency	9	0	−0.14	0.14	0.255	21.10%	0.970
	Medium frequency	7	0.39	0.23	0.55	0	76.60%	0.000
	High frequency	11	0.32	0.19	0.44	0.00	89.00%	0.000
Length of each intervention	Short-duration	3	0.96	0.71	1.21	0.517	0.00%	0.000
	Medium duration	10	0.24	0.11	0.37	0	87.70%	0.000
	Long duration	14	0.07	−0.04	0.18	0.009	53.80%	0.225
Outcomes	ADCS-ADL	4	0.01	−0.17	0.19	0.541	0.00%	0.925
	IADL	10	0.36	0.22	0.51	0.000	83.90%	0.000
	BADL	6	0.31	0.12	0.51	0.000	92.40%	0.002
	ADL	6	0.23	0.05	0.40	0.158	37.30%	0.012
	ADAS-Cog	1	0.12	−0.16	0.40	0	0	0.409

(1) Multi-component training (balance, resistance, aerobic and flexibility training); (2) aerobic exercise; (3) low-intensity training; (4) strength, walking and balance training; (5) resistance training or aerobic exercise.

In summary, the results of the subgroup analysis in this study show significant differences in the impact of different physical exercises on the risk of daily living ability in Alzheimer's patients. Resistance training or aerobic exercise group, long-cycle intervention group (>6 months), medium-frequency intervention group (4–5 times/week), and short duration intervention ( ≤ 30 min) IADL group intervention may have more positive effects on Alzheimer's patients.

## 4 Discussion and conclusion

This study, through systematic review and meta-analysis, included 19 articles encompassing 27 randomized controlled trials (RCTs), primarily investigating the intervention effects of physical exercise on the daily living abilities of Alzheimer's disease (AD) patients. The results indicate that physical exercise can significantly improve the daily living abilities of AD patients, with resistance training or aerobic exercise, long-term interventions, medium-frequency interventions, and short-duration IADL interventions potentially showing better effects.

Physical exercise improves the daily living abilities of AD patients through multiple biological mechanisms. Firstly, subgroup analysis based on intervention content types shows that resistance training or aerobic exercise may have a more positive impact on interventions for AD patients' daily living abilities. Resistance training or aerobic exercise can increase the expression of brain-derived neurotrophic factor (BDNF), thus promoting the survival of hippocampal neurons, synaptic plasticity, and learning and memory abilities in AD patients. This may be related to the increased cardiac output and cerebral blood flow caused by aerobic exercise, which improves brain tissue oxygenation and metabolism (26). Additionally, moderate-intensity aerobic exercise can upregulate the expression of neurotrophic factors such as BDNF and insulin-like growth factor-1 (IGF-1), promoting neurogenesis in the hippocampal region and enhancing synaptic plasticity (27). However, excessively high-intensity aerobic exercise may induce an excessive stress response, leading to elevated cortisol levels and negatively impacting cognitive function. BDNF is an important neurotrophic factor that can activate the tyrosine kinase B (TrkB) receptor, initiating downstream signaling pathways such as phosphatidylinositol 3-kinase (PI3K), protein kinase B (Akt), and mitogen-activated protein kinase (MAPK), promoting neuronal growth, differentiation, and survival (28). Studies have found that aerobic exercise can upregulate BDNF expression levels, enhance synaptic transmission efficiency in the hippocampal region, and improve spatial learning and memory abilities in AD mouse models (29).

In addition to the neuroprotective role of BDNF, recent studies have also highlighted the role of exercise in modulating neuroinflammation, which is increasingly recognized as a key contributing factor in AD progression. Regular physical exercise has been shown to downregulate pro-inflammatory cytokines such as TNF-α and IL-6, while upregulating anti-inflammatory mediators like IL-10, thereby reducing neuroinflammation and promoting a neuroprotective environment (53, 54). This dual mechanism—enhancing neuroplasticity via BDNF and reducing neuroinflammation—may explain the significant improvements in cognitive function and ADL performance observed in AD patients following structured physical exercise interventions.

Secondly, the subgroup analysis of intervention duration revealed that long-term interventions (>6 months) were more effective than short-term and medium-term interventions. This suggests that the improvement in daily living abilities of AD patients through physical exercise may have a time-dependent effect. Some studies have found that a 1-year physical exercise intervention was more effective in improving cognitive function in AD patients compared to a 6-month intervention (30). This may be related to adaptive changes in brain structure and function induced by long-term physical exercise. Animal experiments have shown that long-term aerobic exercise can increase the density of dendritic spines in pyramidal neurons in the hippocampal region of rodents, improving spatial learning and memory abilities (31). Furthermore, long-term physical exercise can delay the progression of brain atrophy in AD patients, especially in memory-related brain regions such as the hippocampus and entorhinal cortex (32). In summary, resistance training for >6 months can activate the glycogen synthase kinase-3β (GSK-3β) pathway, reduce amyloid-β (Aβ) deposition, and alleviate AD pathological changes. GSK-3β is a serine/threonine protein kinase that plays a key role in the pathogenesis of AD. Overactivation of GSK-3β can cause excessive phosphorylation of tau protein, leading to the formation of neurofibrillary tangles and accelerating neuronal apoptosis (33). Additionally, activated GSK-3β can promote the expression of β-site APP-cleaving enzyme 1 (BACE1), accelerating Aβ production (34). Resistance training or aerobic exercise can inhibit GSK-3β activity, reducing tau protein phosphorylation levels and Aβ deposition, thereby delaying AD progression (35).

Thirdly, subgroup analysis of intervention frequency indicated that medium-frequency interventions (4–5 times/week) were most effective. This may be related to the interaction between neural adaptation induced by medium-frequency exercise and exercise intensity. Some studies have found that moderate-intensity aerobic exercise three times a week is more effective in improving executive function in AD patients compared to low-intensity exercise five times a week (36). Another study showed that high-intensity resistance training twice a week had comparable effects on overall cognitive function improvement in AD patients as moderate-intensity resistance training three times a week (37). This suggests that when formulating exercise prescriptions, it is necessary to balance exercise intensity and frequency to select the optimal combination. Choosing physical exercise with appropriate intervention frequency can upregulate the expression of insulin-degrading enzyme (IDE), accelerating Aβ clearance. IDE is a zinc-dependent metalloprotease that can degrade monomeric and oligomeric forms of Aβ, playing an important role in maintaining Aβ homeostasis in the brain (38). Research has found that physical exercise with appropriate intervention frequency can activate peroxisome proliferator-activated receptor-γ (PPAR-γ), thereby upregulating IDE expression, accelerating Aβ clearance, and improving cognitive function in AD patients (39).

Fourthly, subgroup analysis of single intervention duration showed that short-duration interventions ( ≤ 30 min) were most effective. This may be related to the general decline in physical strength and poor fatigue tolerance in AD patients. Prolonged single exercise sessions may cause fatigue in AD patients, reducing their motivation and adherence to exercise. Therefore, when formulating exercise prescriptions for AD patients, the principle of “multiple short sessions” should be followed, avoiding overly long single exercise sessions. However, some studies have found that moderate-intensity aerobic exercise for 60 min per session is more effective in improving executive function in MCI patients compared to 30-min sessions (40). This suggests that the choice of single exercise duration may need to consider factors such as the patient's cognitive function status and exercise tolerance. Single exercise sessions of ≤ 30 min can also improve cerebral blood perfusion in AD patients, reducing ischemic and hypoxic damage to brain tissue. AD patients generally have impaired cerebrovascular function, manifested as reduced cerebral blood flow, decreased vascular reactivity, and increased blood-brain barrier permeability (41). These pathological changes may be related to Aβ deposition in cerebrovascular walls and mediation of inflammatory responses (42). Physical exercise can promote cerebrovascular angiogenesis, increase capillary density, and improve blood perfusion in brain tissue (43). Additionally, physical exercise can upregulate the expression of nitric oxide (NO) synthase, increasing the release of NO from vascular endothelial cells, dilating cerebral blood vessels, and reducing cerebrovascular resistance (44).

Lastly, the subgroup analysis of outcome indicators in this study found that resistance training or aerobic exercise, long-term interventions (>6 months), medium-frequency interventions (4–5 times/week), and short-duration ( ≤ 30 min) IADL interventions were more effective. This suggests that when formulating exercise prescriptions for AD patients, factors such as exercise type, duration, frequency, and single session length should be comprehensively considered. Some studies have found that resistance training conducted for 6 months, 3 times a week, with 45-min sessions, can significantly improve overall cognitive function in AD patients. This may be related to muscle contractions induced by resistance training stimulating the activity of motor neurons and promoting neural connections between the motor cortex and skeletal muscles (45). On the other hand, resistance training can also increase glucose uptake and utilization by skeletal muscles, improving insulin resistance, thereby indirectly enhancing cognitive function in AD patients (46).

Furthermore, the role of insulin resistance in AD pathology is becoming increasingly recognized, and physical exercise has been shown to improve insulin sensitivity, thereby reducing the progression of cognitive decline (52). This is particularly important in the context of AD, as insulin resistance is associated with increased Aβ accumulation and tau hyperphosphorylation, both of which are hallmark features of the disease. Accordingly, interventions that target both metabolic and cognitive functions, such as resistance training, may offer a dual benefit in managing AD. Physical exercise can regulate inflammatory responses in AD patients, reducing levels of pro-inflammatory cytokines such as interleukin-1β (IL-1β), interleukin-6 (IL-6), and tumor necrosis factor-α (TNF-α). Chronic inflammatory response is a crucial factor in AD pathogenesis, accelerating Aβ deposition and tau protein phosphorylation, leading to synaptic dysfunction and neuronal loss (47). IL-1β, IL-6, and TNF-α are key pro-inflammatory factors involved in AD pathological processes, activating microglia and astrocytes, triggering neurotoxic reactions (48). Physical exercise can inhibit the activation of the nuclear factor-κB (NF-κB) signaling pathway, downregulating the expression of inflammatory mediators and alleviating neuroinflammation (49). Additionally, physical exercise can increase levels of anti-inflammatory cytokines such as interleukin-10 (IL-10) and transforming growth factor-β (TGF-β) in AD patients, exerting neuroprotective effects (50).

In conclusion, this study's results elucidate that physical exercise, as a non-pharmacological treatment approach, has advantages such as simple operation, easy acceptance, and low cost, making it an important auxiliary measure in managing AD patients. Multiple studies have shown that symptom-improving drugs like piracetam and donepezil, although capable of temporarily improving cognitive function in AD patients, cannot delay disease progression or reduce mortality risk (51). However, physical exercise may act through multiple molecular mechanisms, including increasing BDNF expression, inhibiting GSK-3β activity, upregulating IDE and NO synthase expression, and regulating inflammatory responses, ultimately improving cognitive dysfunction and enhancing daily living abilities in AD patients. Nevertheless, these mechanisms have not been fully elucidated and require further basic and clinical research for verification. Future research should also explore the potential synergistic effects of combining physical exercise with pharmacological treatments, as this may further enhance therapeutic outcomes for AD patients (51). Moreover, further studies should aim to refine exercise prescriptions by considering individualized factors such as genetic predispositions, disease stage, and comorbidities, to maximize the therapeutic benefits of exercise interventions in the context of AD.

## 5 Limitations

This study has several limitations: (1) There is considerable heterogeneity among the included studies, with significant differences in patient demographics, specific physical exercise protocols, and choice of assessment indicators; (2) The included studies are all short-term interventions, lacking long-term follow-up data; (3) The study subjects are predominantly patients with mild or moderate AD, with limited data on severe AD patients; (4) Most of the included studies use subjective scales to assess patients' cognitive function and daily living abilities, lacking objective neuroimaging and biomarker indicators; (5) This study did not explore in-depth the specific mechanisms and comparative advantages of different types of physical exercise.

## 6 Future research directions

Although this study systematically reviewed the intervention effects of physical exercise on daily living abilities in AD patients, finding that physical exercise can significantly improve daily living abilities in AD patients, with resistance training or aerobic exercise, long-term, medium-frequency, and short-duration IADL interventions showing better effects, there are still many issues that warrant further exploration: (1) In-depth exploration of the molecular mechanisms by which physical exercise improves patients' cognitive function through regulating multiple AD pathological processes, including Aβ deposition, tau protein phosphorylation, synaptic plasticity, oxidative stress, and mitochondrial function, elucidating its potential role in AD prevention and treatment; (2) Targeted comparison of exercise regimens with different types, intensities, frequencies, and durations to explore optimal exercise prescriptions and develop individualized intervention strategies; (3) Comprehensive assessment of the impact of physical exercise on cognitive function in AD patients at different stages (MCI, mild, moderate, and severe), elucidating its application value throughout AD management; (4) Conduct large-sample, multi-center, long-term follow-up cohort studies to evaluate the impact of regular physical exercise on AD risk, disease progression, and prognosis, providing evidence-based support for developing AD prevention and treatment strategies; (5) Strengthen research on the combined application of physical exercise with other interventions such as medications, cognitive training, and neuromodulation, exploring synergistic and complementary comprehensive management models.

## Data Availability

The datasets generated and analyzed during the current study are available from the corresponding author upon reasonable request.
